# Resistance mechanisms and overcoming strategies for CAR T-cell therapy in B-cell hematologic malignancies

**DOI:** 10.3389/fimmu.2026.1861111

**Published:** 2026-06-11

**Authors:** Pupu Li, Xia Xue, Zisen Zhang

**Affiliations:** 1 Zhengzhou Key Laboratory of Lipid Metabolism and Precision Diagnosis & Treatment in Oncology, Oncology Department, The Fifth Affiliated Hospital of Zhengzhou University, Zhengzhou, China; 2 State Key Laboratory of Metabolic Dysregulation & Prevention and Treatment of Esophageal Cancer, Tianjian Laboratory of Advanced Biomedical Sciences, Zhengzhou, China

**Keywords:** B-cell malignancies, CAR T-cell therapy, hematologic cancers, immunotherapy, overcoming strategies, resistance mechanisms

## Abstract

Chimeric antigen receptor (CAR) T-cell therapy has transformed the treatment landscape for relapsed/refractory (R/R) B-cell malignancies, including B-cell non-Hodgkin lymphoma (B-NHL), B-cell acute lymphoblastic leukemia (B-ALL), and related diseases. However, treatment failure caused by primary or secondary resistance remains a major clinical challenge, thereby limiting long-term efficacy and patient survival. Recent studies have systematically clarified the multidimensional mechanisms underlying resistance to CAR T-cell therapy, which can be broadly classified into tumor-intrinsic factors, CAR T-cell dysfunction, and an immunosuppressive tumor microenvironment (TME). At the same time, innovative strategies to overcome these barriers have rapidly emerged, including multitarget CAR design, metabolic and epigenetic modulation, and microenvironment remodeling. This review summarizes the latest advances in the mechanisms of resistance to CAR T-cell therapy and corresponding therapeutic strategies in B-cell malignancies, and further discusses future perspectives to provide a theoretical basis for optimizing CAR T-cell therapy.

## 1 Current progress in the CAR structure

**Figure 1:**
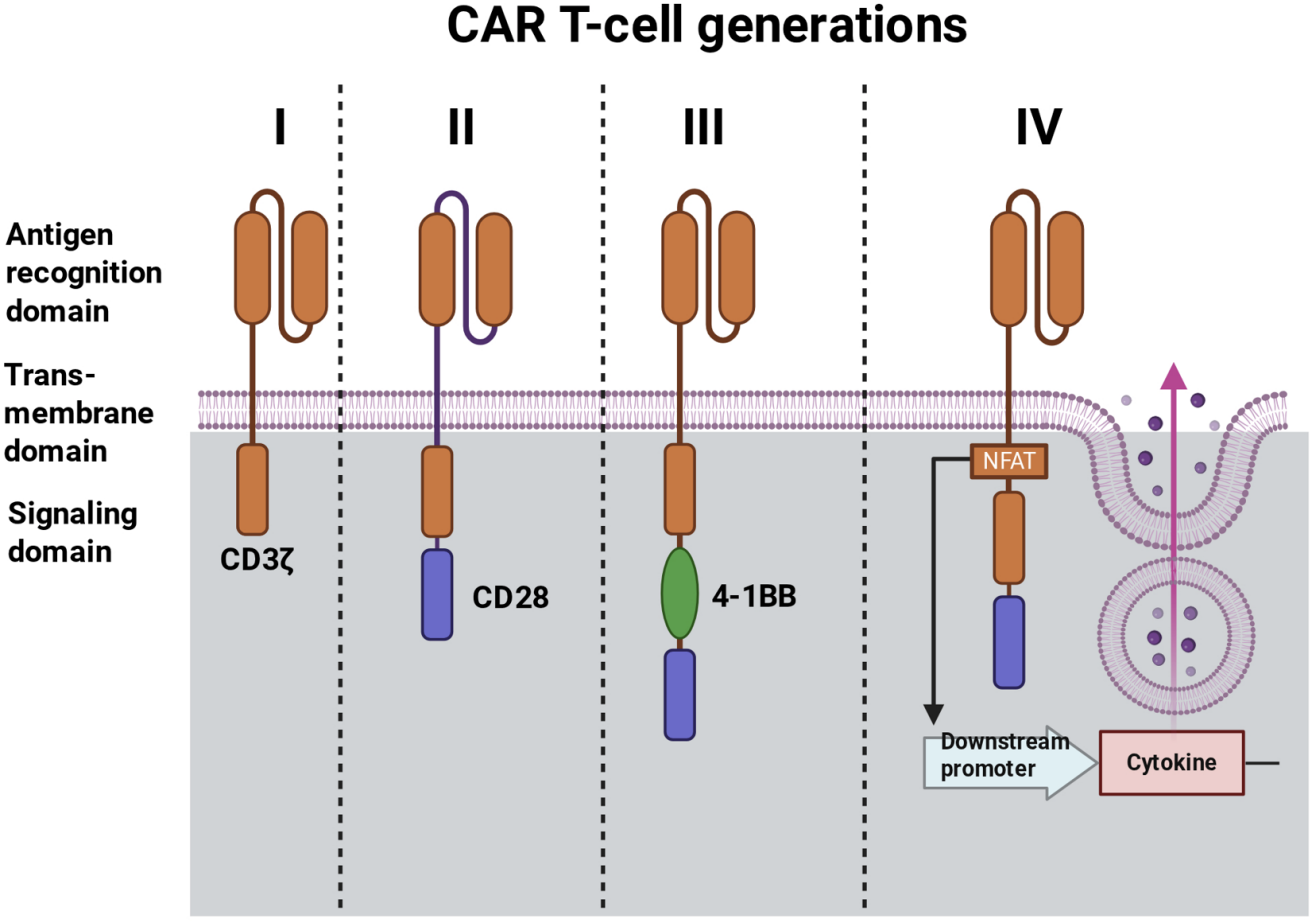
The chimeric antigen receptor (CAR) structure of the four generations. The extracellular antigen-binding domain typically comprises variable heavy and light chains to form a single chain variable fragment (scFv) from a monoclonal antibody. The ectodomain is then coupled with the endodomain through the transmembrane domain. In the first generation, the intracellular domain is typically equipped with the CD3ζ of the T-cell receptor. In the second generation, CD28 or 4-1BB is added to the intracellular domain. The modified third generation then contains both. The novel fourth generation CAR-T cells, known as TRUCKs (T cells redirected for universal cytokine killing), contains genes encoding cytokines or transcription factors, such as IL-12 and IL18, which can enhance activation and function. Created with BioRender.com.

The chimeric antigen receptor (CAR) includes an extracellular single-chain variable fragment (scFv), a transmembrane domain, and an intracellular costimulatory signaling domain (1). CAR-T cells are genetically engineered to directly recognize specific antigens on the surface of tumor cells through the scFv region. The intracellular signaling domain CD3ζ activates T cells by transmitting activation signals. Costimulatory signals enhance the proliferative capacity and durable antitumor activity of CAR-T cells (2). Recognition of tumor antigens by CAR-T cells is not restricted by major histocompatibility complex (MHC) molecules, thereby avoiding immune evasion caused by MHC downregulation in tumor cells (3). The design of CARs has undergone four generations of development (**Figure 1**). First-generation CARs comprise an extracellular antigen-recognition domain and the intracellular signaling domain CD3ζ. Owing to the absence of a costimulatory signaling domain, these CARs cannot sustain prolonged proliferation (4). Second-generation CARs incorporate a single costimulatory domain derived from either CD28 or 4-1BB. Most CAR-T cell therapies approved by the US Food and Drug Administration (FDA) are second-generation products, which have achieved a complete remission rate of more than 90% in patients with relapsed/refractory B-cell malignancies (5).

Although third-generation CAR-T cells offer theoretical advantages, the complexity of their signaling pathways may lead to unintended T-cell exhaustion or overactivation (6). Fourth-generation CAR-T cells, known as TRUCKs (T cells redirected for universal cytokine killing), secrete cytokines or transcription factors to enhance activation, function, and survival (7, 8). In addition, immune checkpoint inhibitors (9, 10) and metabolism-related enzymes (11) have also been incorporated into the design of fourth-generation CARs. In summary, with the close integration of basic research and clinical translation, an increasing number of CAR-T cell products are expected to be approved for clinical application.

## 2 Application of CAR T-cell therapy in B-cell malignancies

CAR-T cells have revolutionized the field of cancer treatment and are considered one of the major medical breakthroughs of the past few decades (12). On July 1, 2014, the US FDA granted Breakthrough Therapy designation to CTL019, a CD19-directed CAR-T cell therapy developed by the University of Pennsylvania. This approval marked the emergence of the first personalized cell therapy for cancer (13). Currently, seven CAR-T cell products have been approved by the US FDA. Among them, Kymriah (14), Yescarta (15), Tecartus (16), Breyanzi (17) and Aucatzyl (18) are used for the treatment of relapsed/refractory (R/R) large B-cell lymphoma (LBCL), R/R B-cell acute lymphoblastic leukemia (B-ALL), R/R mantle cell lymphoma (MCL), and R/R diffuse large B-cell lymphoma (DLBCL), respectively. Abecma (19) and Carvykti (20) are approved for the treatment of relapsed/refractory multiple myeloma (R/R MM) (**Table 1**).

**Table 1:** Currently, FDA-approved CAR T-cell therapies are available worldwide.

Target	Brand name	Cell product	Indications	Company	Approval date
CD19 (14)	Kymriah	Tis-cel	R/R B-ALL in patients ≤ 25 years	Novartis	2017/8
CD19 (15)	Yescarta	Axi-cel	R/R LBCL R/R FL	Gilead Sciences	2017/10
CD19 (16)	Tecartus	Brex-cel	R/R MCL	Gilead Sciences	2020/7
CD19 (17)	Breyanzi	Liso-cel	R/R LBCL	Bristol Myers Squibb	2021/2
BCMA (19)	Abecma	Ide-cel	R/R MM	Bristol Myers Squibb	2021/3
BCMA (20)	Carvykti	Cilta-cel	R/R MM	Legend Biotech	2022/2
CD19 (18)	Aucatzyl	Obe-cel	R/R B-ALL in adults	Autolus Therapeutics	2024/11

R/R, relapsed or refractory; B-ALL, B cell acute lymphoblastic leukemia; LBCL, large B-cell lymphoma; FL, follicular lymphoma; MCL: mantle cell lymphoma; multiple myeloma: MM; Tis-cel, Tisagenlecleucel; Axi-cel, Axicabtagene ciloleucel; Brex-cel, Brexucabtagene autoleucel; Liso-cel, lisocabtagene maraleucel; Ide-cel, Idecabtagene vicleucel; Cilta-cel, Ciltacabtagene autoleucel; Obe-cel, Obecabtagene Autoleucel.

In recent years, clinical trials have shown that CD19-CAR-T cells exhibit substantial therapeutic efficacy in patients with B-cell malignancies (**Table 2**). In the ELIANA study, 81% of patients with B-ALL achieved an overall remission rate with tisagenlecleucel (Tisa-cel) (14). In the registration study of DLBCL, namely the JULIET trial, the complete remission rate was 39% (21). In the ZUMA-1 trial, the complete remission rate was 58% (22), 33% of the relapses were CD19 negative (23). In the ZUMA-2 trial, 68% of patients with MCL achieved complete remission (24), whereas patients with follicular lymphoma (FL) in the ZUMA-5 study had a complete remission rate of 74% (25). In the ZUMA-3 study, the complete remission rate in adults was 56% (26). The ZUMA-8 clinical trial showed that only 27% of patients with chronic lymphocytic leukemia (CLL) experienced CAR-T cell expansion, and only 7% achieved complete remission (27). Recent studies have confirmed that the TOX-NR4A signaling axis specifically regulates CAR-T cell differentiation, metabolic homeostasis and long-term antitumor function, and may be a core molecular pathway driving the exhaustion and therapeutic heterogeneity in CLL-derived CAR-T cells (28). In the ZUMA-7 trial, 65% of patients with LBCL achieved complete remission (29), whereas patients with LBCL in the ZUMA-12 study had a complete remission rate of 78% (30). In the ELARA study, 69% of patients with FL achieved an overall remission rate with Tisa-cel (31). The Pedi CART19 clinical trial showed that 90% of patients with B-ALL achieved complete remission (32). In the AT101 study, 75% of patients with B-NHL achieved an overall remission rate (33). In contrast, B-ALL appears to be more responsive to CAR-T cell therapy (34). Nevertheless, the above−mentioned CAR−T cell products have failed to reproduce such striking clinical success when applied to solid tumors. Solid tumors construct a spatially confined, structurally rigid, and metabolically hostile TME that universally restricts the trafficking, infiltration, metabolic fitness, and persistence of CAR−T cells (35).

**Table 2:** Clinical trials of CD19-CAR T-cell therapy in B-cell malignancies.

Clinical trial	NCT	Histology	Numbers	CART19 product	Costimulatory signals	ORR/CR	PFS
ELIANA (14)	NCT02435849	B-ALL	75	Tis-cel	4-1BB	ORR:81%; CR:60%	73% at 6 months; 50% at 12 months
JULIET (21)	NCT02445248	DLBCL	111	Tis-cel	4-1BB	ORR:52%;CR:39%	65% at 12 months
ZUMA-1 (22)	NCT02348216	LBCL, DLBCL, PMBCL	111	Axi-cel	CD28	ORR:82%;CR:58%	49% at 6 months; 44% at 12 months; 41% at 15 months
ZUMA-2 (24)	NCT02601313	MCL	74	KTE-X19	CD28	ORR:93%;CR:68%	61% at 12 months
ZUMA-3 (26)	NCT02614066	B-ALL	71	KTE-X19	CD28	ORR:71%;CR:56%	58% at 6 months
ZUMA-5 (25)	NCT03105336	FL, MZL	104	Axi-cel	CD28	ORR:92%;CR:74%	/
ZUMA-7 (29)	NCT03391466	LBCL	170	Axi-cel	CD28	CR:65%;	41% at 24 months
ZUMA-12 (30)	NCT03761056	LBCL	40	Axi-cel	CD28	ORR:89%;CR:78%	75% at 12 months
TRANSCEND NHL 001 (17)	NCT02631044	BCL	269	Liso-cel	4-1BB	ORR:73%;CR:53%	51.4% at 6 months; 44.1% at 12 months
ELARA (31)	NCT03568461	FL	98	Tis-cel	4-1BB	ORR:86.2%;CR:69%	/
Pedi CART19 (32)	NCT01626495	B-ALL	30	CTL019	4-1BB	CR:90%	67% at 6 months
AT101 (33)	NCT05338931	B-NHL	12	h1218-CART19	4-1BB	ORR:91.7%;CR:75%	75% at 9.3 months
ZUMA-8 (27)	NCT03624036	B-CLL	15	Brexu-cel	CD28	ORR:47%;CR:7%	/

B-ALL, B cell acute lymphoblastic leukemia; LBCL, large B-cell lymphoma; BCL, B-cell lymphoma; FL, follicular lymphoma; MCL: mantle cell lymphoma; NHL, non-Hodgkin lymphoma; CLL, chronic lymphocyte leukemia; Tis-cel, Tisagenlecleucel; Axi-cel, Axicabtagene ciloleucel; Brex-cel, Brexucabtagene autoleucel; Liso-cel, lisocabtagene maraleucel; Ide-cel, Idecabtagene vicleucel; Cilta-cel, Ciltacabtagene autoleucel; Obe-cel, Obecabtagene Autoleucel. CR, complete response; ORR, overall response rate; PFS, progression-free survival.

## 3 Mechanisms of resistance to CAR T-cell therapy in B-cell malignancies

**Figure 2:**
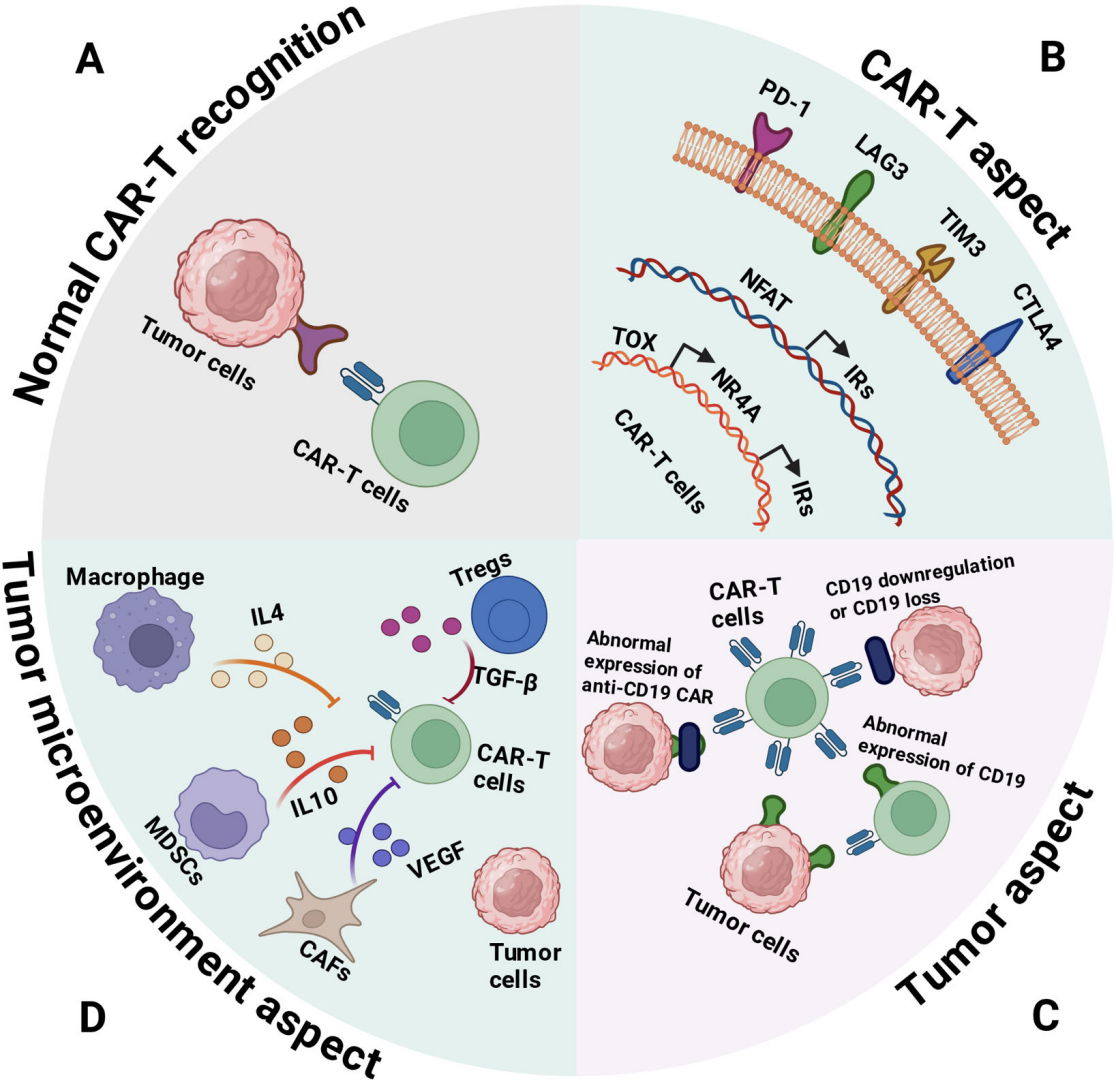
Mechanisms of tumor resistance to CAR T-cell therapy. **(A)** CAR-T cells mediate antitumor effects under normal circumstances. **(B)** Intrinsic mechanisms of resistance to CAR T-cell therapy. **(C)** Tumor cell-intrinsic mechanisms of resistance to CAR T-cell therapy: downregulation or loss of CD19 expression can render cancer cells resistant to CD19-CAR T-cell therapy; because the CD19-CAR binds to the CD19 antigen and subsequently masks the CD19 epitope, CAR-T cells cannot attack cancer cells; the CD19 antigen is transiently transferred to CD19-CAR T cells, which leads to CAR T-cell fratricide and exhaustion. **(D)** Tumor microenvironment-mediated mechanisms of resistance to CAR T-cell therapy. Created with BioRender.com.

Although CAR T-cell therapy has demonstrated remarkable efficacy in B-cell malignancies (36, 37), the lack of response and high relapse rates remain major challenges in clinical research (38, 39). Therefore, investigating the mechanisms underlying resistance to CAR T-cell therapy in B-cell malignancies is essential for improving therapeutic efficacy. This study discusses the factors contributing to resistance to CAR T-cell therapy from three perspectives (**Figures 2A–D**): CAR-T cell-related factors, tumor cell-related factors, and TME -related factors.

### 3.1 CAR-T cell aspect

#### 3.1.1 T-cell defects

Patients with tumors inherently exhibit certain T-cell functional deficiencies, and CAR-T cells generated from these T cells therefore display impaired function (40). Immune dysfunction is a hallmark of CLL and is driven primarily by impaired T-cell function and an increased proportion of regulatory T cells (Tregs) (41, 42). Although CD19-CAR-T cells can induce complete remission in more than 90% of patients with B-ALL (43), only 26% of patients with CLL achieve a durable antitumor response. This discrepancy is likely attributable to the functional status of autologous T cells (44, 45).

#### 3.1.2 Expansion capacity, persistence and cytotoxicity

Expansion capacity, persistence, and cytotoxicity are critical determinants of CAR-T cell efficacy. Early relapse in patients with B-ALL typically occurs within the first few months after remission induced by CAR-T cell therapy and has been shown to be associated with the limited persistence of CAR-T cells (43). CAR-T cells incorporating different types of costimulatory signals exhibit distinct functional characteristics (12, 46). CD28-based CAR-T cells show limited persistence. Gardner et al. demonstrated that patients who experience antigen-positive relapse due to poor CAR-T cell persistence still show loss of CAR-T cell durability after reinfusion (47). Age-related factors also affect the cytotoxicity, persistence, memory phenotype, and naïve T-cell phenotype of CAR-T cells. In elderly patients, CAR-T cells have shorter survival and lower expansion capacity. This may explain the poorer prognosis after CAR-T cell therapy in elderly patients and the higher incidence of antigen-positive relapse. CAR-T cells with a terminally differentiated phenotype have limited longevity compared with stem-like memory T cells. In addition, murine scFvs in CAR constructs may trigger immune rejection, thereby reducing CAR-T cell persistence. Thus, the *in vivo* expansion, persistence, and cytotoxicity of CAR-T cells are closely associated with therapeutic efficacy.

#### 3.1.3 T-cell dysfunction

A critical driver of CAR-T cell exhaustion is persistent antigen exposure (48). Clinical trials have shown that CD28-based CAR-T cells are more prone to exhaustion than 4-1BB-based CAR-T cells (48). Researchers find that 4-1BB signaling links to an increase in mitochondrial function and a memory-like T cell phenotype, leads to increased OXPHOS dependence. GLUT-1 and phosphorylation of mTOR (phosphor-mTOR) were significantly higher in CD28-CAR-T cells, which drives increased dependence on glycolysis (49). Antigen-independent clustering of CAR-scFv induces CAR-CD3ζ phosphorylation, which leads to early exhaustion of CAR-T cells (50). Deficiency of the c-Jun/c-Fos AP-1 heterodimer contributes to T-cell exhaustion and dysfunction (51). Activation of immune checkpoints in the TME induces T-cell exhaustion and is a major barrier to antitumor immunotherapy. T cells are induced to express multiple inhibitory receptors (IRs), including programmed cell death protein 1 (PD-1), lymphocyte activation gene 3 (LAG-3), T-cell immunoglobulin and mucin-domain containing 3 (TIM-3), and cytotoxic T-lymphocyte-associated protein 4 (CTLA-4) (52). PD-1 inhibits T-cell proliferation through binding to PD-L1/PD-L2, suppresses the production of functional molecules such as IFN-γ and IL-2, and limits T-cell survival (35). Studies have shown that PD-1 expression in CAR-T cells increases from the time of reinfusion to the peak expansion phase *in vivo* (53). CTLA-4 inhibits T-cell proliferation and activation by binding to the costimulatory molecules CD80 and CD86 (54). Exhausted T cells also express multiple transcription factors, such as nuclear factor of activated T cells (NFAT) and the thymocyte selection-associated high mobility group box protein (TOX)-NR4A axis, which drive T-cell exhaustion and promote the expression of multiple IRs (34). Epigenetic modifications further drive the stable expression of exhaustion-related transcription factors, leading to irreversible functional impairment. In patients with diffuse large B-cell lymphoma (DLBCL), a higher proportion of LAG-3^+^ CAR-T cells is associated with poorer therapeutic efficacy (55). In patients with CLL receiving CD19-CAR-T cell therapy, transcriptome sequencing revealed that genes significantly upregulated in CAR-T cells from nonresponders were primarily enriched in the exhaustion and apoptosis pathways (45).

### 3.2 Tumor cell aspect

CD19, a transmembrane glycoprotein belonging to the immunoglobulin superfamily, is nearly ubiquitously expressed in B-cell malignancies but is absent in non-B cells, making it an ideal therapeutic target (56). CD19 is retained during malignant transformation and drives oncogenic signaling in various malignancies, including DLBCL, B-ALL, CLL, and follicular lymphoma (FL). Although CD19-CAR-T cells have achieved high objective response rates (ORRs) in the treatment of B-cell malignancies, nonresponse or relapse after remission remains a major clinical challenge (14). Based on alterations in target antigens, the resistance mechanisms of B-cell malignancies to CAR-T cell therapy can be classified into two main categories: antigen-positive relapse and antigen-negative relapse.

#### 3.2.1 Antigen-positive relapse

Antigen-positive relapse is driven by mechanisms that enable cancer cells to evade CAR-T cell-mediated killing despite continued antigen expression. Marco Ruella and colleagues reported that impaired death receptor signaling pathways and elevated expression of antiapoptotic molecules reduce tumor cell sensitivity to CAR-T cell-induced killing (57). Persistent exposure of resistant tumor cells to T cells further leads to T-cell dysfunction. Tumor cells overexpress inhibitory ligands that bind to receptors on CAR-T cells, thereby suppressing their function (57). BCL-2 is upregulated in B-cell malignancies and contributes to tumor cell resistance to CAR-T cell therapy (58). Another important cause of tumor recurrence is the presence of a small subset of tumor cells with stem cell-like properties, which possess strong differentiation and self-renewal capacities and can resist CAR-T cell-mediated killing (59, 60). Deletion of the PTEN gene activates the PI3K/AKT pathway, promotes tumor cell proliferation and survival, and contributes to antigen-positive relapse or resistance (61). Combining CAR-T cell therapy with a pan-AKT inhibitor may therefore improve patient outcomes (61).

#### 3.2.2 Antigen-negative relapse

Although CAR-T cell therapy has revolutionized the treatment of B-cell malignancies, antigen-negative relapse represents the major category of treatment failure or relapse in B-ALL, accounting for approximately 30%-60% of relapsed cases (62). Antigen-negative relapse is a common pattern of recurrence in childhood ALL (62) and accounts for approximately one-third of relapsed cases of large B-cell lymphoma (63). The mechanisms underlying antigen-negative relapse mainly involve the following processes: preexisting target antigen-negative tumor cells, epigenetic silencing of CD19 expression under therapeutic pressure, target antigen loss mediated by gene mutations, alternative splicing variants or lineage switching, and impaired antigen presentation (64–69). A study from the Children’s Hospital of Philadelphia collected data from 628 patients with B-ALL. Approximately 7% of patients exhibited typical CD19-negative expression, and 24% exhibited low-normal expression, which prevented CAR-T cells from effectively recognizing tumor antigens and led to relapse (70). Orlando and colleagues identified frameshift insertions and deletions in exons 2–5 of the CD19-coding sequence in patients who relapsed after CAR-T cell therapy, resulting in loss of surface CD19 expression (71).

A 20-year-old patient with B-ALL relapsed 9 months after receiving CD19-CAR-T cell therapy. The relapsed cells lacked CD19 but expressed CAR proteins, suggesting accidental CAR transduction into leukemia cells during T-cell manufacturing. The CAR product bound in cis to the CD19 epitope on the B-cell surface, thereby masking the antigen, enabling immune evasion from CAR-T cell recognition, and conferring resistance (72). In CD19-negative patients with relapsed ALL, the leukemia cell surface marker CD81 is absent. Loss of CD81 prevents the processing and maturation of CD19 in the Golgi apparatus (64, 72). CAR-T cells also exhibit limited immune activity against tumor cells with low target antigen density. In patients with B-ALL who relapse after CD22-CAR-T cell therapy, CD22 is either absent or markedly reduced, highlighting the critical role of target antigen density in determining CAR-T cell efficacy (73).

Hamieh et al. reported that CARs induce reversible antigen loss through trogocytosis, an active process in which target antigens are transferred to T cells, thereby reducing target density on tumour cells and diminishing T cell activity by promoting fratricidal T cell killing and exhaustion (74). Thus, it directly impairs CAR−T cell survival and anti−tumor function. Antigen-negative relapse after CAR-T cell therapy arises from tumor plasticity and immune evasion. Therefore, multitarget CAR-T cell therapy is of critical importance for improving prognosis.

### 3.3 Tumor microenvironment aspect

#### 3.3.1 Immunosuppressive cells

The TME is composed of various cell types, including myeloid-derived suppressor cells (MDSCs), tumor-associated macrophages (TAMs), regulatory T cells (Tregs), and cancer-associated fibroblasts (CAFs) (34). The TME interferes with the function and infiltration of effector T cells, thereby promoting tumorigenesis (34), and serves as a critical factor influencing the efficacy of CAR-T cell therapy (75). In treatment-resistant Hodgkin lymphoma, tumor cells convert macrophages into immunosuppressive TAMs, thereby suppressing T-cell proliferation (76). In patients with relapsed/refractory B-cell non-Hodgkin lymphoma (B-NHL) receiving CD19-CAR-T cell therapy, TAM infiltration was negatively correlated with prognosis (77). Tumor-derived granulocyte-macrophage colony-stimulating factor (GM-CSF) promotes STAT3-mediated induction of PD-L1 expression on MDSCs, which inhibits T-cell antitumor responses through binding to PD-1 on CAR-T cells (78). In a sarcoma mouse model, MDSCs directly suppressed the proliferation and function of GD2-CAR-T cells (79). Studies have shown that an increased number of tumor-infiltrating Tregs is negatively correlated with patient prognosis (80). In a study using blinatumomab, responders had fewer Treg cells than nonresponders (81). Multiple myeloma is characterized by an immunosuppressive TME and abundant CAFs, which promote tumor cell growth and suppress CAR-T cell function (82).

#### 3.3.2 Tumor cell metabolism

By depriving effector lymphocytes of nutrients such as glucose, lipids, and amino acids, tumor cells in the TME create an environment characterized by hypoxia, acidic pH, and high levels of immunosuppressive metabolites. Indoleamine 2,3-dioxygenase (IDO) is highly expressed in hematologic malignancies. It mediates the conversion of the essential amino acid tryptophan into the immunosuppressive metabolite kynurenine, inhibits T-cell proliferation, induces apoptosis, and is negatively correlated with the prognosis of patients (83). Ninomiya et al. reported that CD19-CAR-T cells can inhibit the growth of IDO-negative tumor cells but have no effect on IDO-positive tumor cells (84). Gene expression profiling of pancreatic ductal adenocarcinoma (PDA) cells resistant to tMUC1-CAR-T cells revealed high expression of IDO and cyclooxygenase (COX) (85). Tumor cells catabolize the semi-essential amino acid arginine to promote their proliferation, and the resulting arginine-depleted microenvironment impairs CAR-T cell proliferation (11). Adenosine produced by tumor cells binds to adenosine receptors on the surface of immune cells such as cytotoxic T cells, thereby activating intracellular inhibitory signaling and suppressing the proliferation, function, and antitumor activity of CAR-T cells (86). In patients with tumors, both hypoxia and glucose depletion lead to lactate accumulation, which decreases pH in the TME, reduces the secretion of IL-2 and IFN-γ, and suppresses effector T-cell function (87). Hypoxia is caused by excessive oxygen consumption by tumor cells and disorganization of the tumor microvasculature. Hypoxia-inducible factor (HIF) upregulates PD-L1 expression on tumor cells and TAMs, thereby inhibiting CD8^+^ T cells (88). Inflammatory factors in the TME, such as prostaglandin E_2_ (PGE_2_), can induce Foxp3 expression in CD4^+^ T cells, thereby enhancing Treg function and weakening the antitumor immune response (89).

## 4 Strategies for overcoming resistance to CAR T-cell therapy in B-cell malignancies

### 4.1 Enhancing CAR T-cell antigen recognition ability

**Figure 3:**
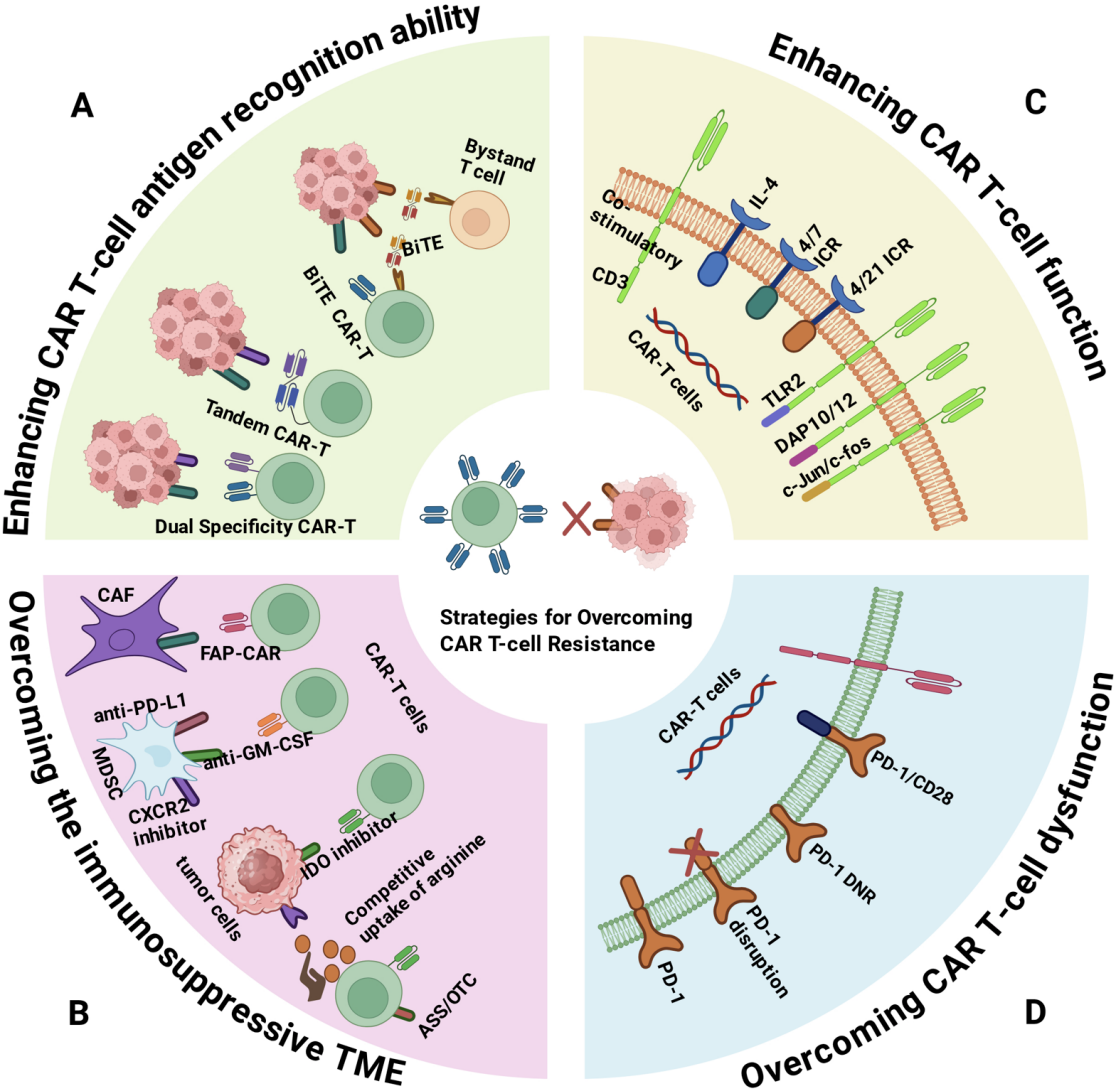
Strategies to overcome resistance to CAR T-cell therapy in B-cell malignancies. **(A)** Enhancing CAR T-cell antigen recognition ability: Dual targeting methods include bispecific CAR-T cells, which include 2 different CARs targeting 2 antigens per T-cell; tandem CAR-T cells with 2 antigen targets on each CAR; gene-editing CAR-T cells to secrete bispecific T-cell engagers (BiTEs), that is BiTEs typically consist of two single-chain antibodies, one specific for tumor-associated antigens (TAAs) and the other specific for CD3, mediating the formation of synapses between tumor cells and T cells. **(B)** Overcoming the immunosuppressive TME: Other factors contributing to resistance are the presence of immunosuppressive cell types (such as MDSCs and CAFs) and immunosuppressive ligands (such as PD-L1 and GM-CSF) in the TME. The amino acid arginine is required for T cell activation. Low arginine levels in the TME create a deficient metabolic environment that resulting in resistance to CAR T-cell therapy. CAR-T cells overexpressing ASS/OTC compete with tumor cells for the uptake of arginine. **(C)** Enhancing CAR T-cell function: Incorporating costimulatory molecules, such as TLR2 and DAP10/12, into the 3′ end of CAR augments costimulatory signaling in CAR-T cells; ectopic overexpression of c-Jun/c-fos results in transcriptional rewiring that renders T cells exhaustion resistant; the inhibitory endodomain of the IL-4 receptor can be replaced by activator receptors, including IL-7 and IL-21, to transduce prosurvival signals to lymphocytes; **(D)** Overcoming CAR T-cell dysfunction: Chimeric switch receptors (CSRs), such as PD-1/CD28, chimeric receptor fusing inhibitory extracellular domains with stimulatory intracellular domains, which redirects immunosuppressive signals into T−cell−activating signal; Dominant-negative receptors (DNRs), truncated inhibitory receptor lacking intracellular signaling domains, which blocks suppressive signaling via competitive ligand sequestration; Knocking out the PD-1 gene using gene-editing technology, such as CRISPR/Cas9, can block the suppression signal in CAR-T cells. Created with BioRender.com.

Numerous studies have shown that target antigen-negative relapse is the major category of CAR T-cell therapy failure or recurrence in patients with B-ALL. To prevent immune escape of tumor cells and improve antigen recognition by CAR-T cells, dual-targeted or multi-targeted CAR-T cells that simultaneously target two or more distinct tumor antigens (90, 91), as well as combinations of multiple single-antigen-targeted CAR-T cell products, can enhance antitumor activity and reduce recurrence. Sequential therapy using CAR-T cells directed against different antigens has also been applied clinically (**Figure 3A**). CD19/CD22 CAR-T cell therapy has demonstrated favorable safety and efficacy in patients with B-ALL and LBCL (91, 92). CD19/CD20 CAR-T cells have been shown to improve the complete remission rate in patients with B-cell lymphoma (93). For tumor cells to evade CAR-T cell recognition and killing, simultaneous loss of both target antigens is required, thereby reducing the likelihood of immune escape and improving the efficacy of CAR T-cell therapy.

Engineering CAR-T cells to secrete bispecific T-cell engagers (BiTEs) represents another strategy to enhance the antigen-recognition capacity of CAR-T cells. BiTEs typically consist of two single-chain antibodies, one specific for a tumor-associated antigen (TAA) and the other specific for CD3, which mediate the formation of immunological synapses between tumor cells and T cells. Blinatumomab, a CD19/CD3 bispecific T-cell engager, activates endogenous T cells, promotes T-cell proliferation, and induces directed lysis of CD19-positive cells (94). It has been approved by the US FDA for the treatment of B-ALL (95).

### 4.2 Overcoming the immunosuppressive TME

Research indicates that the immunosuppressive TME is a major factor limiting CAR T-cell function. The TME mainly comprises immunosuppressive cells, metabolic constraints, inflammatory cytokines, and immune checkpoints. Immunosuppressive MDSCs inhibit the proliferation and function of GD2-CAR T cells (79), whereas eradication of MDSCs with all-trans retinoic acid (ATRA) can enhance the antitumor efficacy of GD2-CAR T cells (79). In the TME, Burga et al. neutralized granulocyte-macrophage colony-stimulating factor (GM-CSF) and PD-L1 on MDSCs, thereby abolishing their immunosuppressive effects and improving the antitumor activity of CAR T cells (**Figure 3B**). The combination of CAR-T cells with MDSC-targeted therapies provides a rationale for future clinical trials (78). Cancer-associated fibroblasts (CAFs) are major components of the TME and promote tumor cell growth in multiple myeloma. To overcome CAF-induced suppression of CAR-T cells, dual targeting of CAFs and tumor cells with CAR-T cells can markedly reverse TME-induced immunosuppression (82).

Ninomiya et al. reported that pretreatment of tumor-bearing mice with fludarabine and cyclophosphamide reduced IDO expression in tumor cells and enhanced the efficacy of CD19-CAR T cells in xenograft lymphoma models (84). Cancer cells with high metabolic demands compete with lymphocytes for nutrients, thereby suppressing T-cell function (96). Tumor cells promote their own proliferation by degrading arginine, and the resulting arginine-depleted environment impairs CAR T-cell function. Studies have shown that T cells can be re-engineered to express the arginine resynthesis enzymes argininosuccinate synthase (ASS) and ornithine transcarbamylase (OTC) together with different chimeric antigen receptors. This enzymatic modification enhances CAR-T cell proliferation without causing loss of cytotoxicity or aggravating exhaustion. *In vivo*, enzyme-modified CAR-T cells have shown relatively effective leukemia cell elimination (11).

### 4.3 Enhancing CAR T-cell function

The *in vivo* expansion, persistence, and antitumor activity of CAR-T cells are closely associated with therapeutic efficacy. Therefore, one strategy to overcome tumor resistance to CAR T-cell therapy is to enhance the proliferation, persistence, and cytotoxicity of CAR-T cells *in vivo*. Patients with tumors exhibit inherent functional deficiencies in their T cells, and CAR-T cells generated from these T cells also display functional impairment (97). The successful development of allogeneic CAR-T cells from healthy donors may address issues related to T-cell defects and manufacturing challenges, such as insufficient T-cell numbers or poor T-cell function (39). Research has shown that increasing the proportion of less differentiated T-cell subsets enhances the *in vivo* expansion capacity and persistence of CAR-T cells (45). CD28-based CAR-T cells exhibit stronger expansion capacity than CAR-T cells containing the 4-1BB costimulatory domain (98). In contrast, the 4-1BB domain promotes fatty acid oxidation and increases the central memory phenotype, thereby improving CAR T-cell persistence (98, 99). 4-1BB-based CAR-T cells not only show greater persistence but also alleviate T-cell exhaustion caused by sustained CAR signaling (100). Replacing CD28 with 4-1BB as the costimulatory signal can shift CAR-T cells from an exhausted state toward a memory-like state (101), reducing the expression of exhaustion markers and upregulating pathways related to the negative regulation of hypoxia and apoptosis.

Toll-like receptor 2 (TLR2) is highly expressed on activated and memory T cells and enhances T-cell expansion and cytokine production (102). Incorporation of the TLR2 receptor domain into the CAR structure can improve the expansion capacity, persistence, and antitumor response of CAR-T cells (103) (**Figure 3C**). The intracellular domains of DAP10/12 can serve as signaling motifs for CARs, enabling unimpaired production of huLym-1-B CAR-T cells with potent antitumor efficacy (104). Exhausted T cells may result from a relative deficiency of the c-Jun/c-Fos AP-1 heterodimer, and c-Jun overexpression can render CAR-T cells resistant to exhaustion (51). Interleukin-7 (IL-7) promotes the generation of memory-like lymphocytes, such as central memory T (Tcm) cells and stem cell memory T (Tscm) cells, which persist longer *in vivo* and exhibit sustained antitumor activity (105–107). IL-15 is a T-cell growth factor, and CAR-T cells expressing IL-15 display increased expansion capacity and antitumor activity (108). CAR-T cells capable of secreting IL-21 exhibit improved expansion capacity and increased IFN-γ secretion (109). CAR-T cells that secrete IL-18 show increased *in vivo* expansion and persistence, together with the ability to modulate the tumor microenvironment (TME) and amplify effective endogenous antitumor immune responses (110). Soluble inhibitory molecules in the TME, such as interleukin-4 (IL-4) and interleukin-10 (IL-10), suppress the proliferation and cytotoxicity of CAR-T cells. CAR-T cells expressing inverted cytokine receptors (ICRs) can counteract these inhibitory molecules, resulting in enhanced persistence and antitumor efficacy (106).

### 4.4 Overcoming CAR T-cell dysfunction

During target-cell recognition by CAR-T cells, the immunosuppressive molecule PD-1 is upregulated. Activation of the PD-1/PD-L1 pathway negatively regulates CAR T-cell function. Blocking the PD-1/PD-L1 pathway can enhance CAR T-cell cytotoxicity and overcome exhaustion. Several novel strategies have been proposed. Combination therapy with CAR-T cells and immune checkpoint inhibitors (**Figure 3D**): PD-1-blocking antibodies promote CAR T-cell expansion and enhance the antitumor response in patients with progressive lymphoma after CD19-CAR T-cell therapy (111). Genetically engineered exhaustion-resistant CAR-T cells: CRISPR/Cas9-mediated knockout of PD-1 in CAR-T cells improves their activity, with encouraging results reported in early clinical trials (112, 113). Genetically engineered CAR-T cells capable of secreting PD-1 antibodies: modified CAR-T cells express the scFv segment of PD-1 antibodies, thereby blocking the interaction between PD-1 and PD-L1. Compared with PD-1 monoclonal antibody therapy, PD-1 antibody-secreting CAR-T cells enable more precise tumor targeting while minimizing off-target toxicity in other tissues (114). In addition, genetically engineered CAR-T cells can express endogenous PD-1 dominant-negative receptors (DNRs) (115): truncated inhibitory receptor lacking intracellular signaling domains, which blocks suppressive signaling via competitive ligand sequestration, thereby limiting CAR T-cell exhaustion (116). Another approach involves engineering CAR-T cells with PD-1/CD28 fusion receptors, namely the Chimeric Switch Receptor (CSR): chimeric receptor fusing inhibitory extracellular domains with stimulatory intracellular domains, which redirects immunosuppressive signals into T−cell−activating signals, thereby significantly enhancing the therapeutic efficacy of CAR-T cells (117). Moreover, histone deacetylase (HDAC) inhibitors and DNA methyltransferase (DNMT) inhibitors can reverse CAR-T cell exhaustion by regulating the expression of exhaustion-related genes, such as TOX and PD-1. Targeting immune checkpoint signaling pathways can significantly enhance the efficacy of CAR-T cells in the treatment of malignant tumors and improve patient prognosis.

## 5 Summary and prospects

Chimeric antigen receptor (CAR) T-cell therapy has led to substantial advances in the treatment of relapsed/refractory B-cell malignancies. Nevertheless, resistance to CAR T-cell therapy remains a critical challenge that limits long-term survival. Studies have shown that intrinsic T-cell defects (such as exhaustion and metabolic dysfunction), adaptive changes in tumor cells (such as antigen loss and low antigen density), and hostile microenvironmental factors (such as immunosuppressive cytokines and nutrient deprivation) interact and mutually reinforce each other, ultimately leading to treatment failure. These interconnected pathways highlight the necessity of adopting combination strategies to overcome resistance. Over the past decade, multidimensional strategies have been developed to overcome these barriers, including multitarget CAR design, combination therapies with immune checkpoint inhibitors, epigenetic modulators, or small-molecule drugs, as well as approaches targeting the immunosuppressive TME to restore CAR T-cell effector function. Furthermore, the integration of precision epigenetics, synthetic biology, and single-cell omics technologies may facilitate the development of smarter and more robust CAR T-cell products, improve response durability, reduce toxicity, and broaden their applicability across a range of B-cell malignancies. Meanwhile, *in vivo* generation of CAR-T cells can simplify the manufacturing process, reduce ex vivo manipulation, and attenuate toxicity. Precise gene disruption using gene-editing technologies can specifically disrupt exhaustion-associated genes, thereby enhancing CAR-T cell persistence while minimizing off-target toxicity. Ultimately, through continuous innovation and multidisciplinary collaboration, CAR T-cell therapy may be further optimized to achieve durable remission and consolidate its role as a potentially curative strategy for B-cell malignancies.
