# Correction: Resistance mechanisms and overcoming strategies for CAR T-cell therapy in B-cell hematologic malignancies

**DOI:** 10.3389/fimmu.2026.1907428

**Published:** 2026-07-08

**Authors:** Pupu Li, Xia Xue, Zisen Zhang

**Affiliations:** 1 Zhengzhou Key Laboratory of Lipid Metabolism and Precision Diagnosis & Treatment in Oncology, Oncology Department, The Fifth Affiliated Hospital of Zhengzhou University, Zhengzhou, China; 2 State Key Laboratory of Metabolic Dysregulation & Prevention and Treatment of Esophageal Cancer, Tianjian Laboratory of Advanced Biomedical Sciences, Zhengzhou, China

**Keywords:** B-cell malignancies, CAR T-cell therapy, hematologic cancers, immunotherapy, overcoming strategies, resistance mechanisms

Affiliation “Zhengzhou Key Laboratory of Lipid Metabolism and Precision Diagnosis & Treatment in Oncology, Oncology Department, the Fifth Affiliated Hospital of Zhengzhou University, Zhengzhou, Henan, China” was omitted for author “Pupu Li, Zisen Zhang”. This affiliation has now been added for author “Pupu Li, Zisen Zhang”.

The original version of this article has been updated.

The **References** for “32, 23 , 24, 31, 33, 26, 27, 28, 29, 30” was erroneously written as

“32. Massimo M, Virginia N, Barbara L, Filippo Antonio C, Marta P, Caterina A, et al. Chimeric antigen receptor T-cell therapy: what we expect soon. (2022) 23:13332. doi: 10.3390/ijms232113332

23. Michael W, Javier M, Andre G, Frederick LL, Caron AJ, Brian TH, et al. Three-year follow-up of KTE-X19 in patients with relapsed/refractory mantle cell lymphoma, including high-risk subgroups, in the ZUMA-2 study. (2022) 41:555–67. doi: 10.1200/jco.21.02370

24. Bijal DS, Michael RB, Olalekan OO, Aaron CL, Maria RB, William BD, et al. KTE-X19 anti-CD19 CAR T-cell therapy in adult relapsed/refractory acute lymphoblastic leukemia: ZUMA-3 phase 1 results. (2021) 138:11–22. doi: 10.1182/blood.2020009098

31. Davids M, Kenderian S, Flinn I, Hill B, Maris M, Ghia P, et al. ZUMA-8: a phase 1 study of brexucabtagene autoleucel in patients with relapsed/refractory chronic lymphocytic leukemia. (2025) 146:938–43. doi: 10.1182/blood.2024027460

33. Joyce C, Isaac FL-M, Hyungseok S, Chan-Wang JL, Laura JH, Takashi S, et al. NR4A transcription factors limit CAR T cell function in solid tumours. (2019) 567:530–4. doi: 10.1038/s41586-019-0985-x

26. Frederick LL, David BM, Caron AJ, Miguel-Angel P, Marie-José K, Olalekan OO, et al. Axicabtagene ciloleucel as second-line therapy for large B-cell lymphoma. (2021) 386:640–54. doi: 10.1056/nejmoa2116133

27. Sattva SN, Michael D, Javier M, Matthew LU, Catherine T, Olalekan OO, et al. Axicabtagene ciloleucel as first-line therapy in high-risk large B-cell lymphoma: the phase 2 ZUMA-12 trial. (2022) 28:735–42. doi: 10.1038/s41591-022-01731-4

28. Fowler N, Dickinson M, Dreyling M, Martinez-Lopez J, Kolstad A, Butler J, et al. Tisagenlecleucel in adult relapsed or refractory follicular lymphoma: the phase 2 ELARA trial. (2022) 28:325–32. doi: 10.1038/s41591-021-01622-0

29. Allison Barz L, Haley N, Yimei L, Hongyan L, Regina M, Amanda D, et al. CD19-targeted chimeric antigen receptor T-cell therapy for CNS relapsed or refractory acute lymphocytic leukaemia: a *post-hoc* analysis of pooled data from five clinical trials.

(2021) 8:e711–22. doi: 10.1016/s2352-3026(21)00238-6

30. Yunlin Z, Ruchi PP, Ki Hyun K, Hyungwoo C, Jae-Cheol J, Seong Hyun J, et al. Safety and efficacy of a novel anti-CD19 chimeric antigen receptor T cell product targeting a membrane-proximal domain of CD19 with fast on- and off-rates against non-Hodgkin lymphoma: a first-in-human study. (2023) 22:200. doi: 10.1186/s12943-023-01886-9

It should be

“23, 24, 26, 27, 28, 29, 30, 31, 32, 33”

“23. Massimo M, Virginia N, Barbara L, Filippo Antonio C, Marta P, Caterina A, et al. Chimeric antigen receptor T-cell therapy: what we expect soon. (2022) 23:13332. doi: 10.3390/ijms232113332

24. Michael W, Javier M, Andre G, Frederick LL, Caron AJ, Brian TH, et al. Three-year follow-up of KTE-X19 in patients with relapsed/refractory mantle cell lymphoma, including high-risk subgroups, in the ZUMA-2 study. (2022) 41:555–67. doi: 10.1200/jco.21.02370

26. Bijal DS, Michael RB, Olalekan OO, Aaron CL, Maria RB, William BD, et al. KTE-X19 anti-CD19 CAR T-cell therapy in adult relapsed/refractory acute lymphoblastic leukemia: ZUMA-3 phase 1 results. (2021) 138:11–22. doi: 10.1182/blood.2020009098

27. Davids M, Kenderian S, Flinn I, Hill B, Maris M, Ghia P, et al. ZUMA-8: a phase 1 study of brexucabtagene autoleucel in patients with relapsed/refractory chronic lymphocytic leukemia. (2025) 146:938–43. doi: 10.1182/blood.2024027460

28. Joyce C, Isaac FL-M, Hyungseok S, Chan-Wang JL, Laura JH, Takashi S, et al. NR4A transcription factors limit CAR T cell function in solid tumours. (2019) 567:530–4. doi: 10.1038/s41586-019-0985-x

29. Frederick LL, David BM, Caron AJ, Miguel-Angel P, Marie-José K, Olalekan OO, et al. Axicabtagene ciloleucel as second-line therapy for large B-cell lymphoma. (2021) 386:640–54. doi: 10.1056/nejmoa2116133

30. Sattva SN, Michael D, Javier M, Matthew LU, Catherine T, Olalekan OO, et al. Axicabtagene ciloleucel as first-line therapy in high-risk large B-cell lymphoma: the phase 2 ZUMA-12 trial. (2022) 28:735–42. doi: 10.1038/s41591-022-01731-4

31. Fowler N, Dickinson M, Dreyling M, Martinez-Lopez J, Kolstad A, Butler J, et al. Tisagenlecleucel in adult relapsed or refractory follicular lymphoma: the phase 2 ELARA trial. (2022) 28:325–32. doi: 10.1038/s41591-021-01622-0

32. Allison Barz L, Haley N, Yimei L, Hongyan L, Regina M, Amanda D, et al. CD19-targeted chimeric antigen receptor T-cell therapy for CNS relapsed or refractory acute lymphocytic leukaemia: a *post-hoc* analysis of pooled data from five clinical trials. (2021) 8:e711–22. doi: 10.1016/s2352-3026(21)00238-6

33. Yunlin Z, Ruchi PP, Ki Hyun K, Hyungwoo C, Jae-Cheol J, Seong Hyun J, et al. Safety and efficacy of a novel anti-CD19 chimeric antigen receptor T cell product targeting a membrane-proximal domain of CD19 with fast on- and off-rates against non-Hodgkin lymphoma: a first-in-human study. (2023) 22:200. doi: 10.1186/s12943-023-01886-9

The original version of this article has been updated.

